# Xenoantigen-Dependent Complement-Mediated Neutralization of Lymphocytic Choriomeningitis Virus Glycoprotein-Pseudotyped Vesicular Stomatitis Virus in Human Serum

**DOI:** 10.1128/JVI.00567-19

**Published:** 2019-08-28

**Authors:** Lisa Pipperger, Iris Koske, Nicole Wild, Brigitte Müllauer, Daniela Krenn, Heribert Stoiber, Guido Wollmann, Janine Kimpel, Dorothee von Laer, Zoltán Bánki

**Affiliations:** a Division of Virology, Medical University of Innsbruck, Innsbruck, Austria; b Christian Doppler Laboratory for Viral Immunotherapy of Cancer, Medical University of Innsbruck, Innsbruck, Austria; Instituto de Biotecnologia/UNAM

**Keywords:** complement, neutralization, oncolytic virus, vesicular stomatitis virus, xenoantigens

## Abstract

Neutralization by antibodies and complement limits the effective dose and thus the therapeutic efficacy of oncolytic viruses after systemic application. We and others previously showed that pseudotyping of oncolytic rhabdoviruses such as maraba virus and vesicular stomatitis virus (VSV) with the lymphocytic choriomeningitis virus glycoprotein (LCMV-GP) results in only a weak induction of neutralizing antibodies. Moreover, LCMV-GP-pseudotyped VSV (VSV-GP) was significantly more stable in normal human serum (NHS) than VSV. Here, we demonstrate that depending on the cell line used for virus production, VSV-GP showed different complement sensitivities in nonimmune NHS. The NHS-mediated titer reduction of VSV-GP was dependent on activation of the classical complement pathway, mainly by natural IgM antibodies against xenoantigens such as galactose-α-(1,3)-galactose (α-Gal) or *N*-glycolylneuraminic acid (Neu5Gc) expressed on nonhuman production cell lines. VSV-GP produced on human cell lines was stable in NHS. However, VSV-GP generated in transduced human cells expressing α-Gal became sensitive to NHS. Furthermore, GP-specific antibodies induced complement-mediated neutralization of VSV-GP independently of the producer cell line, suggesting that complement regulatory proteins potentially acquired by the virus during the budding process are not sufficient to rescue the virus from antibody-dependent complement-mediated lysis. Thus, our study points to the importance of a careful selection of cell lines for viral vector production for clinical use.

**IMPORTANCE** Systemic application aims to deliver oncolytic viruses to tumors as well as to metastatic lesions. However, we found that xenoantigens incorporated onto the viral surface from nonhuman production cell lines are recognized by natural antibodies in human serum and that the virus is thereby inactivated by complement lysis. Hence, to maximize the effective dose, careful selection of cell lines for virus production is crucial.

## Abstract (precis)

Systemic application aims to deliver oncolytic viruses to tumors as well as to metastatic lesions. However, we found that xenoantigens incorporated onto the viral surface from nonhuman production cell lines are recognized by natural antibodies in human serum and that the virus is thereby inactivated by complement lysis. Hence, to maximize the effective dose, careful selection of cell lines for virus production is crucial.

## INTRODUCTION

Systemic treatment with oncolytic viruses (OVs) is a promising approach to treat disseminated tumors in different cancer types (1). Vesicular stomatitis virus (VSV), an oncolytic virus of the *Rhabdoviridae* family, has been demonstrated to be an effective oncolytic agent and vaccine vector platform in clinical trials and animal models (2, 3). However, VSV easily induces a vector-neutralizing antibody response within a few days following administration (4). Furthermore, despite its low seroprevalence in humans, VSV is neutralized in nonimmune human, mouse, and dog sera. This neutralization is related to natural IgMs inducing complement-mediated lysis (CML) (5, 6). Thus, both antibody- and complement-mediated neutralization of VSV could potentially weaken the efficacy of systemic oncolytic therapy of cancer.

The complement system represents an evolutionarily old innate defense mechanism against invading pathogens (7). Activation of the complement system via the classical, lectin, or alternative pathways converges at the third complement component (C3). Direct activation of the complement cascade by pathogens through lectin and alternative pathways or, alternatively, the induction of classical complement pathway by specific IgG and IgM antibodies bound to the microbial surface plays an important role in the host defense against bacteria and viruses. Complement activation increases phagocytosis via opsonization with C3 fragments, promotes inflammation via the generation of complement anaphylatoxins C3a and C5a, and induces direct CML of susceptible pathogens via the generation of the C5b-C9 membrane attack complex.

VSV has two major limitations for therapeutic applications: (i) its neurotoxicity and (ii) the fact that VSV easily induces neutralizing antibody responses (4, 8). Thus, VSV glycoprotein G has been replaced by the glycoprotein GP of LCMV, and the chimeric VSV-GP demonstrates the same oncolytic capacity as VSV (9). More importantly, VSV-GP overcomes both limitations of VSV. VSV-GP is not neurotoxic and does not induce neutralizing antibodies upon the first application (10, 11). Furthermore, VSV-GP has been demonstrated to be more stable in human serum compared to VSV (9).

The systemic delivery of infectious oncolytic virus is critical for clinical efficacy, and thus a careful characterization of virus serum stability of VSV-GP was performed. Our study demonstrates differences in CML of VSV-GP produced in different cell lines, which was dependent on the presence of xenoantigen-specific antibodies in human serum. Xenoantigens are antigens of one species that induce an immune response in members of a different species. Thus, natural antibodies in human serum against xenoantigens derived from nonhuman virus-producing cells might reduce effective dose of OVs.

## RESULTS

### Production cell line-dependent serum stability of VSV-GP.

**FIG 1:**
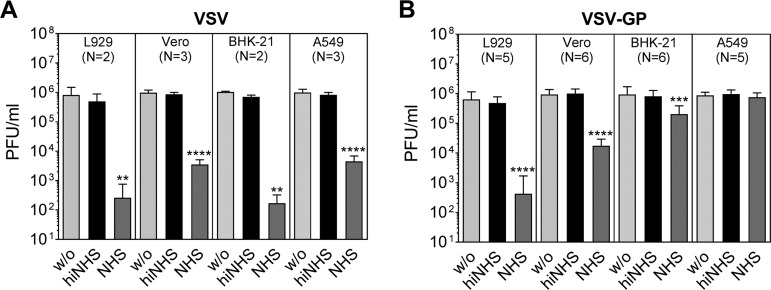
VSV and VSV-GP show different serum sensitivities if produced on different cell lines. VSV (A) and VSV-GP (B) were produced on different cell lines: murine L929, hamster BHK-21, African green monkey Vero, and human A549. Serum resistance assays with 50% NHS were performed with an incubation period of 45 min at 37°C. Plaque titrations were conducted to analyze the remaining titer. GMEM (w/o) and heat-inactivated NHS (hiNHS) served as controls. Mean values with standard deviations (SD) of independent experiments (N) are shown. Data were analyzed by GraphPad Prism software using ANOVA, followed by Dunnett’s multiple-comparison test (****, ***, and **, significant at *P* < 0.0001, *P* < 0.001, and *P* < 0.01, respectively).

First, we confirmed that the titer of VSV is drastically reduced after incubation with nonimmune human serum (NHS) as a source of complement (Fig. 1A) (6). Medium alone (w/o) or heat-inactivated NHS (hiNHS) served as controls. VSV produced on murine L929 or hamster BHK-21 cells showed a titer drop of >3 logs; also, the titer of virus grown on African green monkey Vero cells and human A549 cells was strongly reduced in the serum resistance assay. Interestingly, in contrast to VSV, the serum sensitivity of LCMV GP-pseudotyped VSV-GP was dependent on the producer cell line. Similar to VSV, VSV-GP produced on L929 cells and Vero cells resulted in a significant titer loss of up to 4 and 2 logs, respectively (Fig. 1B). However, VSV-GP derived form BHK-21 cells was relatively stable in NHS with a titer loss up to 1 log compared to VSV, which is in line with previous observations (10). In contrast to VSV, VSV-GP produced on human A549 (Fig. 1B) or HeLa (data not shown) cells was completely resistant to NHS. Furthermore, cell lines used for virus production showed similar serum sensitivities as we observed with VSV-GP produced in the corresponding cell line (data not shown).

### VSV-GP is serum sensitive in a time- and concentration-dependent manner.

**FIG 2:**
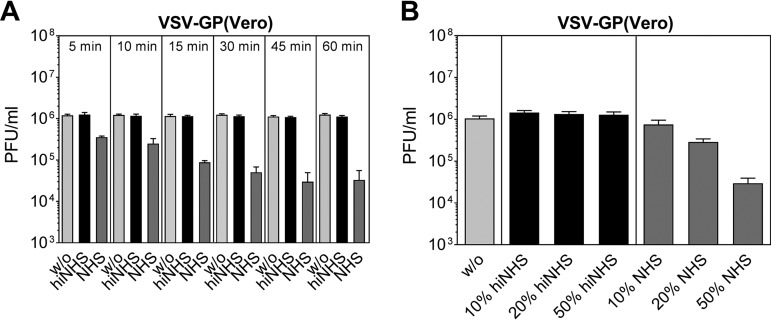
Serum sensitivity of VSV-GP is time and dose dependent. (A) VSV-GP produced on Vero cells was incubated for different periods of time (5 to 60 min) at 37°C with GMEM (w/o), hiNHS, or NHS. (B) VSV-GP(Vero) was incubated for 45 min with different concentrations of NHS or hiNHS (10, 20, or 50%). Afterward, the virus was titrated in plaque assays using BHK-21 cells. Data were generated from two independent experiments with two technical replicates. Mean values are shown, and error bars indicate the SD.

To further characterize the serum-mediated neutralization of VSV-GP, we tested different time periods of incubation in the presence of NHS using VSV-GP produced on Vero cells (Fig. 2A). Already after 5 min, a titer reduction of more than 50% was observed, and after 15 min a >1-log decline of infectious virus titer was detected. After 45 min of exposure time to NHS, a maximal reduction of up to 2 logs of VSV-GP(Vero) was reached. As expected, we could also show that viral neutralization was dependent on the concentration of NHS (Fig. 2B). VSV-GP(Vero) was incubated in the presence of different dilutions of hiNHS or NHS (10, 20, or 50%) for 45 min. The presence of NHS at a dilution of 1:10 resulted in only a slight titer reduction of VSV-GP(Vero). Increasing the concentration of NHS to 20 and 50% led to a reduction of VSV-GP infectious titers to nearly 1 log and up to 2 logs, respectively. Incubation of VSV-GP in the presence of hiNHS did not affect the virus titer.

### Neutralization of VSV-GP in NHS is mediated by the activation of the classical complement pathway.

**FIG 3:**
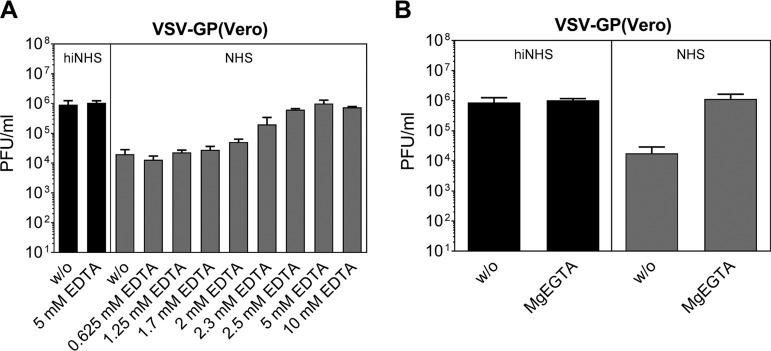
Blockade of complement activation pathways abrogates serum sensitivity of VSV-GP. Serum sensitivity assays with VSV-GP(Vero) were performed in different concentrations of EDTA (from 0.625 to 10 mM) (A) or with 5 mM MgEGTA (B). The remaining infectious virus titers were determined by plaque titration. Three independent experiments with two technical replicates were performed. Mean values are shown, and error bars indicate the SD.

Since heat inactivation of NHS completely abrogated serum neutralization of both VSV and VSV-GP, the serum sensitivity of the viruses is most likely related to CML. To confirm the involvement of complement in serum neutralization of VSV-GP, we used EDTA to block complement activation. Addition of EDTA in a concentration range from 2.5 to 10 mM completely blocked NHS-mediated titer loss of VSV-GP(Vero) (Fig. 3A) and VSV-GP(BHK-21) (data not shown), supporting the role of complement in VSV-GP(Vero) and VSV-GP(BHK-21) serum sensitivity. To define which of the three complement pathways is involved in CML of VSV-GP(Vero), we used magnesium EGTA (MgEGTA) that selectively inhibits the initiation of the classical and the lectin but not the alternative complement pathway (12, 13). As we could completely block CML of VSV-GP(Vero) (Fig. 3B) and VSV-GP(BHK-21) (data not shown) with MgEGTA, the alternative complement pathway is negligible for complement-mediated virus neutralization.

**FIG 4:**
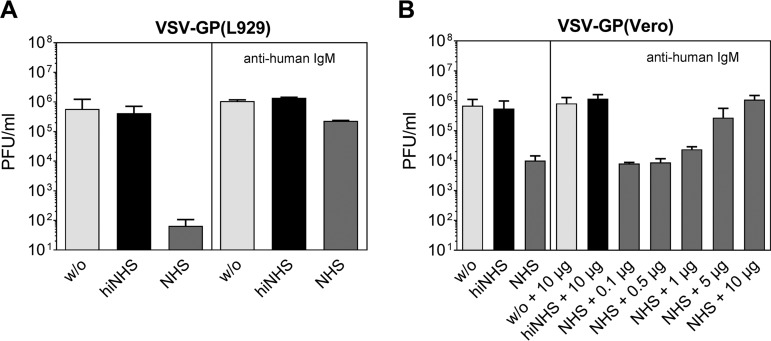
Blocking IgM-mediated complement activation of the classical complement pathway reduces CML of VSV-GP. NHS, hiNHS, or GMEM (w/o) were preincubated with an anti-human IgM antibody (10 μg [A] or the indicated amount [B]) to block the IgM-mediated activation of the classical complement pathway. Subsequently, serum resistance assays were performed using VSV-GP(L929) (A) and VSV-GP(Vero) (B), and viral titers were determined by plaques assays. The data show the results from two independent experiments, with two technical replicates. Mean values are shown, and error bars indicate the SD.

To further investigate CML of VSV-GP, we next inhibited the IgM-induced classical complement activation by preincubating NHS with an anti-human IgM antibody as described previously (6, 14). By blocking IgM-mediated complement activation, the CML of VSV-GP(L929) was strongly reduced (Fig. 4A). If the anti-human IgM antibody with VSV-GP(Vero) was used, a concentration-dependent, complete inhibition of CML could be seen (Fig. 4B).

### Xenoantigens α-Gal and Neu5Gc are involved in the CML of VSV-GP derived from nonhuman cell lines.

**FIG 5:**
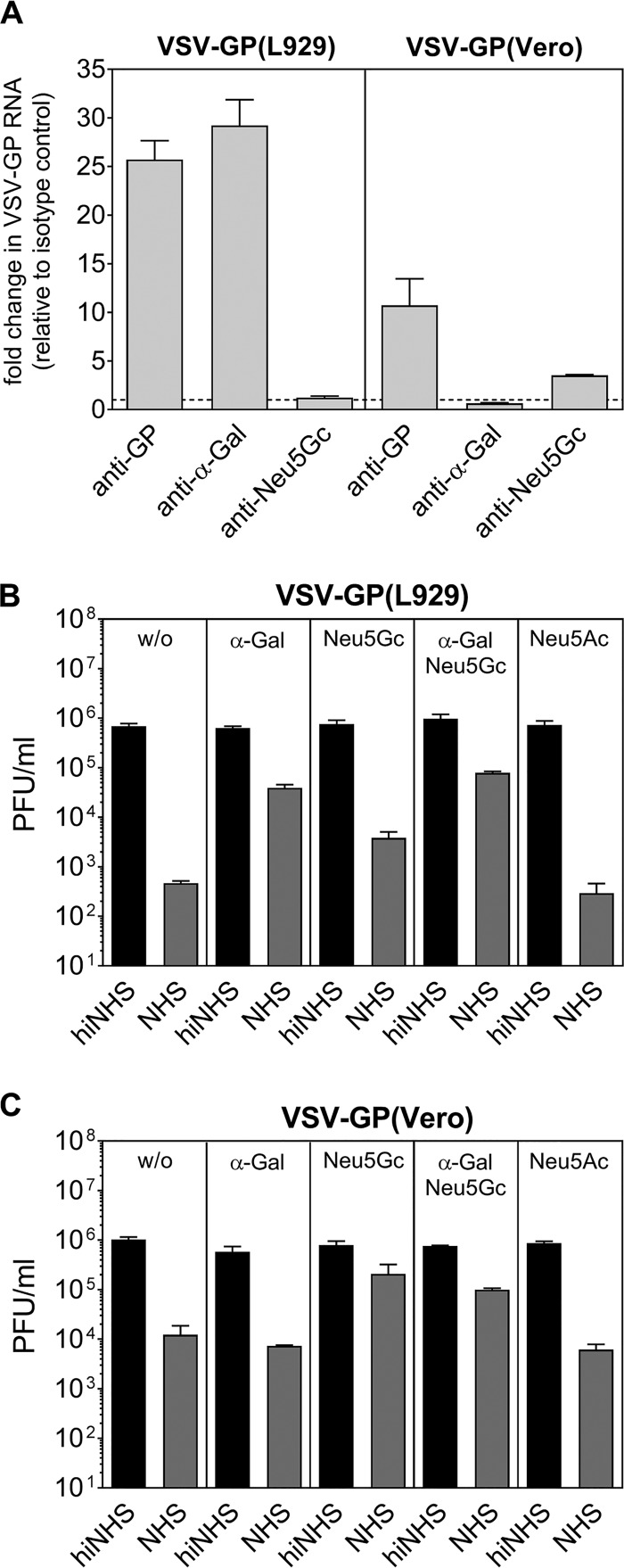
CML of VSV-GP can be reduced by inhibition of natural Abs using soluble α-Gal and Neu5Gc. (A) A virus capture assay using Abs against GP, α-Gal, or Neu5Gc reveals the presence of α-Gal on VSV-GP(L929) and Neu5Gc on VSV-GP(Vero). Sera were preincubated with soluble disaccharide Galα1,3Gal (α-Gal) or sialic acid *N*-glycolylneuraminic acid (Neu5Gc) prior to the addition of viral particles. *N*-Acetylneuraminic acid (Neu5Ac) served as **a** negative control. Serum sensitivity assays of VSV-GP(L929) (B) and VSV-GP(Vero) (C) were performed, and infectious viral titers were determined by plaque assays. The data show the results from two independent experiments with two technical replicates. Mean values are shown, and error bars indicate the SD.

Serum sensitivity of VSV-GP derived from nonhuman cell lines suggest that the classical complement pathway may be activated by natural antibodies (Abs) recognizing xenoantigens. The majority of xenoantigen-recognizing natural Abs (IgMs, IgGs, and IgAs) in human serum are directed against a carbohydrate epitope named Galα1-3Galβ1-4GlcNAc-R (α-Gal) (15, 16). Using a virus capture assay (VCA), we could capture VSV-GP(L929) virions with an α-Gal-specific Ab, revealing the presence of α-Gal on the surface of VSV-GP(L929) (Fig. 5A). To analyze whether this xenoantigen is involved in CML of VSV-GP, we preincubated hiNHS and NHS with the soluble disaccharide Galα1-3Gal (α-Gal) to block anti-α-Gal natural Abs before conducting the serum sensitivity assays. This resulted in 2-log reductions in the CML of VSV-GP derived from mouse L929 cells (Fig. 5B). In contrast, no inhibition of CML by preincubation with α-Gal was observed when VSV-GP(Vero) was used, where the epitope is known to be lacking on the producer cell line (Fig. 5C) (17). Besides α-Gal, other xenoepitopes may be involved in the CML of VSV-GP derived from nonhuman cell lines. Sialic acids differ in the expression on distinct cells and play a role in human complement activation (18, 19). By VCA we could confirm the presence of *N*-glycolylneuraminic acid (Neu5Gc) on the surface of VSV-GP(Vero) virus (Fig. 5A). Next, by preincubation with Neu5Gc, we blocked natural Abs against this epitope, which is expressed on all animal cells except human cells. As a negative control, the most abundant silica acid, expressed also on human cells, *N*-acetylneuraminic acid (Neu5Ac) was used. By blocking Neu5Gc-specific Abs, we could partially inhibit CML of VSV-GP(L929) by up to 1 log (Fig. 5B). The same extent of partial CML inhibition was found for VSV-GP produced on Vero or BHK-21 cells (Fig. 5C and data not shown, respectively). In contrast, Neu5Ac did not affect the CML of VSV-GP.

**FIG 6:**
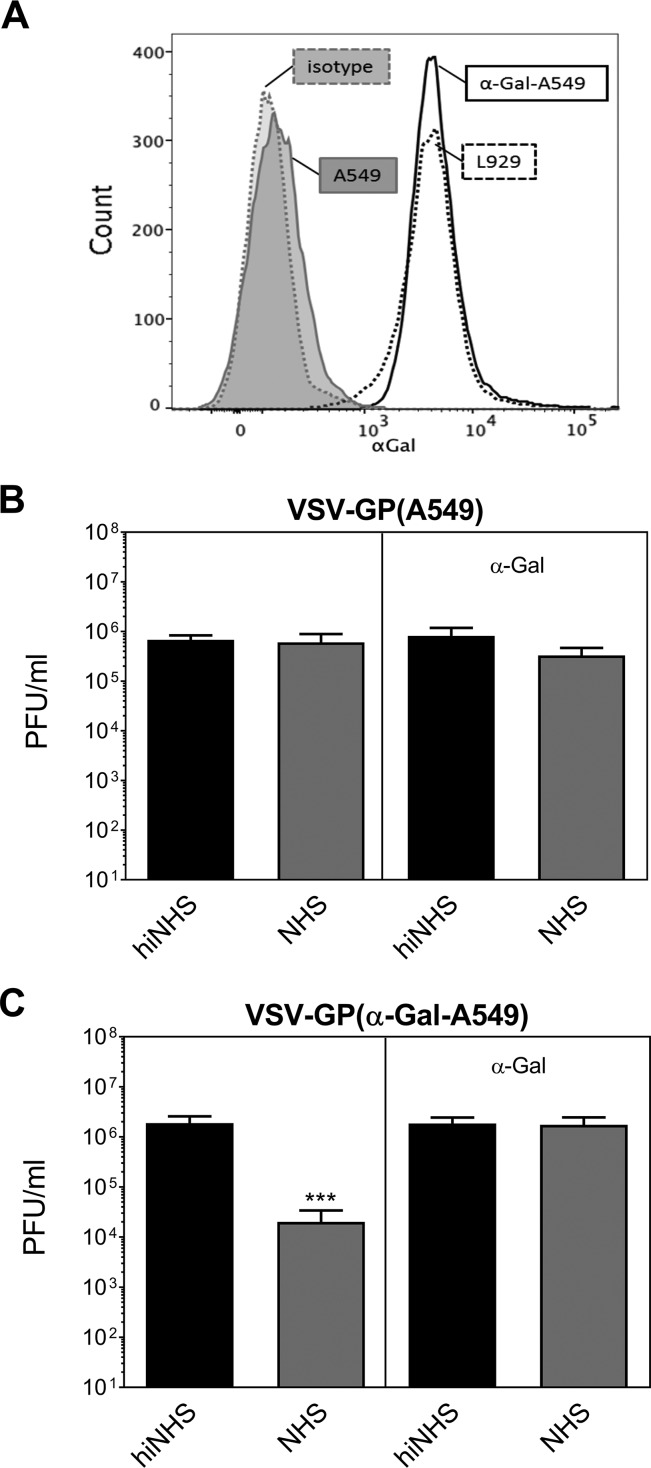
Expression of α-Gal on A549 cells renders VSV-GP serum sensitive. (A) The human cell line A549 was stably transduced with 3Galβ1-GlcNAc-α1-galactosyltransferase (α-GT), which leads to the presence of the α-Gal epitope on the cell surface. The expression of the xenoantigen α-Gal on nontransduced A549 cells and transduced α-Gal-A549 cells was determined with an anti-α-Gal antibody, followed by incubation with a labeled secondary antibody. Subsequently, the cells were analyzed by flow cytometry. As a positive control, mouse L929 cells were used; as a negative control, an appropriate isotype control was used. (B and C) VSV-GP was produced on α-GT**-**transduced cells [VSV-GP(α-Gal A549)]. Serum resistance assays of VSV-GP(A549) (B) and VSV-GP(α-Gal-A549) (C) were performed in the presence or absence of soluble α-Gal. Virus titers were determined by plaque titration. The data show the results of two independent experiments with two technical replicates. Mean values are shown, and error bars indicate the SD. Data were analyzed by GraphPad Prism software using ANOVA, followed by Dunnett’s multiple-comparison test (***, significant at *P* < 0.001).

To finally prove that CML is initiated by α-Gal-specific natural Abs present in human serum, we stably transduced A549 cells with the enzyme 3Galβ1-GlcNAc-α1-galactosyltransferase (α-GT). This enzyme is essential for α-Gal synthesis and is silenced in human cells. A549 cells transduced with α-GT expressed α-Gal (Fig. 6A), which consequently should be incorporated into VSV-GP produced on these cells. Virus grown on nontransduced A549 cells was again serum resistant, and the addition of α-Gal had no effect on serum stability (Fig. 6B). VSV-GP derived from α-GT transduced A549 cells [VSV-GP(α-Gal-A549)] induced CML in the presence of NHS, resulting in a titer reduction of up to 2 orders of magnitude (Fig. 6C). Further, we could block CML of VSV-GP(α-Gal-A549) by adding soluble α-Gal to the assay, thereby demonstrating the specificity of α-Gal-induced CML (Fig. 6C).

### GP-specific Abs and immune serum induce complement-mediated lysis of VSV-GP derived from a human cell line.

**FIG 7:**
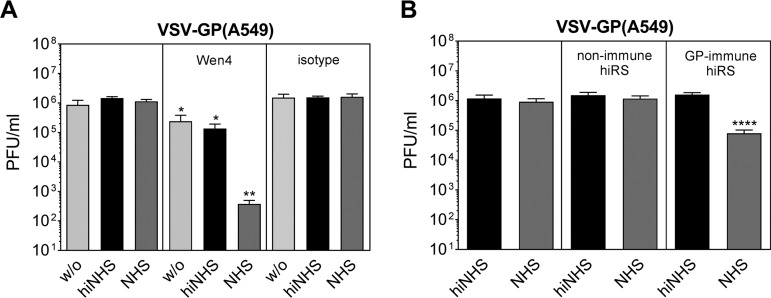
GP-specific antibodies induce CML of VSV-GP. (A) VSV-GP(A549) was preincubated with monoclonal LCMV GP-specific antibody (Wen4) or an appropriate isotype control. Rabbits were vaccinated with VSV-GP, and at 10 days postimmunization sera were collected and heat inactivated (hiRS). (B) Virus was preincubated with nonimmune or GP-immune hiRS for 10 min. Serum sensitivity assays were conducted with NHS, hiNHS, or GMEM (w/o) in two independent experiments with two technical replicates. Mean values are shown, and error bars indicate the SD. Data were analyzed by GraphPad Prism software using ANOVA, followed by Dunnett’s multiple-comparison test (****, **, and *, significant at *P* < 0.0001, *P* < 0.01, and *P* < 0.05, respectively).

VSV-GP(α-Gal-A549) showed serum sensitivity, suggesting that A549-derived virus can be destroyed by complement if activated by Abs. Therefore, it was of interest to study the CML of VSV-GP(A549) in the presence of specific antibodies against the glycoprotein GP. In the absence of complement, classical neutralization of VSV-GP by the GP-neutralizing monoclonal antibody Wen4 (20) reduced the virus titer by about 1 log independent of the producing cell line [Fig. 7A for VSV-GP(A549) and data not shown for VSV-GP(BHK-21)]. If combined with NHS, Wen4 antibodies further decreased the titer of VSV-GP(A549) (Fig. 7A) and VSV-GP(BHK-21) (data not shown) up to 4 logs. In addition to Wen4, we used GP-specific immune sera derived from VSV-GP-vaccinated rabbits collected at day 10 postimmunization. After preincubation of VSV-GP(A549) with heat-inactivated nonimmune or immune rabbit serum as a source of GP-specific Abs, we conducted serum resistance assays with NHS. In contrast to Wen4, rabbit immune sera after VSV-GP immunization did not show classical neutralization capacity in the presence of hiNHS. However, antibody binding induced viral titer reductions of >1 log when NHS was present (Fig. 7B). Thus, VSV-GP induced binding antibodies with the capacity to neutralize VSV-GP in a complement-dependent manner.

## DISCUSSION

VSV-GP represents a promising new therapeutic agent. The virus selectively replicates in tumor cells and shows auspicious results in cancer treatment in various mouse models (10, 21, 22). Furthermore, additional genes can be added to the viral genome, and VSV-GP can be used as a vaccine vector (11, 23). From the clinical point of view, the systemic delivery of OVs might be beneficial not only due to the simplicity of application but also because viruses can reach disseminated tumors or metastatic lesions easily. However, the inactivation of OVs in the bloodstream by neutralizing antibodies and the complement system reduces the efficacy of systemic applications of virotherapies (24, 25). The serum sensitivity of VSV-GP was dependent on the presence of producer cell-derived xenoantigens on the virus recognized by natural IgM antibodies present in human serum. Binding of xenoantigen-specific antibodies induced the classical complement pathway, which in turn resulted in complement-mediated lysis of VSV-GP in nonimmune human serum. In contrast, VSV-GP produced on human cells was stable in NHS.

First, we demonstrated that compared to VSV, VSV-GP is more stable in nonimmune human serum when produced on hamster or human cells but not on mouse or monkey cells. The serum sensitivity of VSV has been shown to be dependent on complement activation by natural antibodies recognizing viral epitopes (5, 6). In contrast to VSV, our data showed that the serum stability of VSV-GP depends on the producer cell line since the virus was completely resistant against NHS when produced on human A549 cells. Blocking complement by heat inactivating NHS or using EDTA or MgEGTA fully abrogated the titer loss of VSV-GP. This provides evidence that the activation of the classical or lectin pathway of complement activation is required for VSV-GP neutralization in NHS. The blockade of IgM-mediated complement activation resulted in an entirely restored VSV-GP(Vero) titer supporting a crucial role of activation the classical complement pathway by naturally occurring IgM antibodies for the serum sensitivity of VSV-GP(Vero). The remaining titer loss of VSV-GP produced on mouse L929 cells after IgM blockade might be related to IgG binding, which can also activate the classical complement pathway (26, 27). Taken together, NHS-mediated neutralization of VSV-GP(L929) and VSV-GP(Vero) suggests a destruction of the virus by complement-mediated lysis (CML). A previous study has provided electron microscopy analysis of VSV in the presence of NHS and showed clear virolysis (28). Ab-mediated CML of VSV-GP relies on the same mechanism described for VSV; thus, we suppose that complement-mediated neutralization is mainly related to virolysis. However, we could not exclude that a steric hindrance through the deposition of complement proteins on the viral surface is also involved in the neutralization.

During the budding process, VSV-GP virions may incorporate cellular membrane components, as already shown for other enveloped viruses which can potentially be recognized by antibodies (29). Highly abundant natural Abs (IgMs and IgGs) in human serum target the disaccharide α-Gal (30). The lack of α-GT in Old World monkeys, apes, and humans leads to the absence of α-Gal on cells of these species (17). According to this, in a VCA we could demonstrate the presence of α-Gal on VSV-GP produced on L929 but not on Vero cells. In subsequent experiments, we showed that complement activation by natural α-Gal-specific antibodies in NHS strongly reduces the titer of VSV-GP produced on mouse L929 cells. The weaker serum sensitivity of VSV-GP(BHK-21) can be explained by the lack of α-Gal on BHK-21 cells (31). Previous data on other enveloped viruses, such as VSV, specific herpesviruses, and type C retroviruses, which are neutralized in NHS depending on α-Gal availability in infected cells, support our findings (30–32, 49). VSV-GP produced on Old World monkey Vero cells was also strongly sensitive to NHS despite the lack of α-Gal expression. However, blocking of *N*-glycolylneuraminic acid-specific antibodies significantly reduced the CML of VSV-GP(Vero). In line with this result, Neu5Gc was detected on VSV-GP(Vero) cells using a VCA. The remaining neutralization of VSV-GP(Vero) in NHS indicates that Abs against xenoantigens other than *N*-glycolylneuraminic acid are most likely involved in the induction of the classical complement pathway (33).

Both xenoantigen- and GP-specific antibody-dependent complement-mediated neutralization of VSV-GP might be important for *in vivo* systemic virus applications. Whereas xenoantigen-specific Ab-dependent CML is dominant after the first application, GP-specific Abs become relevant upon repeated applications of VSV-GP. In previous studies, we have shown that although VSV-GP weakly induces GP-specific neutralizing Ab responses (at least in mice), a boostable production of Abs against antigens encoded by the virus can be observed (11, 34). Antibody-dependent CML is most likely enhanced after second and third applications of VSV-GP when both xenoantigen- and GP-specific antibodies are presented and even boosted. Thus, the presence of both types of Abs results in an enhanced Ab deposition on the viral surface hence an increased induction of classical complement activation and subsequent virolysis. The relative contribution of xenoantigen- and GP-specific Abs in viral neutralization is difficult to differentiate and might be dependent on both the concentration and the affinity of such Abs in individual serum samples. A previous study has shown that GP-specific antibodies induce a robust complement-mediated neutralization which decreases the effective dose of LCMV GP-pseudotyped maraba virus delivered in tumors *in vivo* (25). That study clearly demonstrates that Ab-dependent complement-mediated neutralization of OVs reduces systemic delivery *in vivo*. Since the CML by xenoantigen- or GP-specific Abs relies on the same mechanism of complement activation it is likely that xenoantigen-specific antibodies also reduce the effective dose *in vivo* similarly to GP-specific antibody neutralization. However, this aspect is difficult to investigate *in vivo*. There are xenoantigens, which are known to react with antibodies present in nonimmune human serum. In contrast, there are, to our knowledge, no similar xenoantigens that are useful in experimental animal models. Furthermore, the mouse, representing the easiest model for *in vivo* investigations, is not suitable since mouse complement is less efficient compared to human complement due to its lower lytic activity (35). Because of these difficulties, we could not provide direct *in vivo* relevance of our findings in the present report, and this aspect needs to be investigated in future studies.

Our results support the use of a human producer cell line to allow efficient systemic delivery of VSV-GP and most likely also of other enveloped oncolytic viruses in humans. Nevertheless, it was shown in different animal models that LCMV GP-specific antibodies arise after repeated virus applications (25). Accordingly, we noticed a complement-dependent neutralization of VSV-GP in the presence of GP-specific antibodies. Furthermore, the expression of α-Gal on α-GT-transduced human A549 cells leads to CML of VSV-GP produced on these cells. This indicates that serum resistance of VSV-GP(A549) is due to the lack of complement activation rather than an inhibition of CML by human complement regulatory proteins (CRPs) potentially acquired on the viral surface from human producer cells during the budding process. Viruses like herpesvirus, retrovirus, and mumps virus, as well as VSV, can assemble CRPs onto their surfaces, and it was shown that this incorporation can lead to a reduction of complement-mediated lysis (28, 36, 37). We could detect CRPs such as CD55, CD46, and CD59 on VSV-GP(A549) viral particles (data not shown). However, our data suggest that the presence of these CRPs is not sufficient to protect the VSV-GP from CML, when antibodies either against xenoepitopes or viral epitopes are present.

Several approaches have already been tested to rescue the complement-mediated serum inactivation of VSV. The insertion of selective mutations into the glycoprotein, the overexpression of surface CRPs or PEGylation of VSV all improved the stability in human serum but did not result in a complete rescue of CML (38–40). Thus, the generation of a serum-resistant VSV remains challenging. The importance of serum stability for the effective delivery of viral vectors was shown in different studies demonstrating that the depletion of complement *in vivo* led to a higher treatment efficiency (24, 25, 41, 42). Here, we showed that in contrast to VSV, VSV-GP is resistant to CML in NHS when produced on human cells, at least in the absence of virus-specific Abs. With respect to the systemic delivery of VSV-GP, as an oncolytic agent, the presence of xenoantigens on the virus surface derived from nonhuman production cells is expected to be highly disadvantageous. In contrast, when VSV-GP is used as a vaccine vector xenoantigens such as α-Gal might increase the immunogenicity of the vaccine (43). Thus, we point out that the producer cell line for viral vectors matters and has to be carefully chosen in consideration of the application purpose.

## MATERIALS AND METHODS

### Ethics statement.

Animal experiments were performed according to the national animal experimentation law and were approved by the ethics committees of the Medical University of Innsbruck and the Austrian Federal Ministry of Science and Research (BMWF-66.011/0010-WF/V/3b/2016).

### Cells.

The mouse connective tissue fibroblast line L929 (ACC 2, from DSMZ), the hamster kidney fibroblast line BHK21 (clone 13, from ECACC), the African green monkey kidney epithelial line Vero (CCL-81, from ATCC), and the human lung carcinoma line A549 (ACC 107, from DSMZ) were used in our experiments. L929 and A549 cells were cultured in Dulbecco modified Eagle medium (Lonza) supplemented with 10% fetal calf serum (FCS; Life Technologies) and 4 mM l-glutamine (from Gibco/Thermo Fisher). BHK-21 cells were maintained in Glasgow minimal essential medium (GMEM; Lonza) supplemented with 10% FCS (Life Technologies), 5% tryptose phosphate broth (Gibco/Thermo Fisher), and 4 mM l-glutamine. Vero cell were cultivated under serum-free conditions in VP-SFM medium (Gibco/Thermo Fisher) supplemented with 4 mM l-glutamine.

### Viruses.

VSV-GP, a VSV pseudotyped with the glycoprotein (GP) of LCMV is described elsewhere (10). VSV and VSV-GP were produced on L929, BHK-21, Vero, and A549 cells. Virus supernatants were filtered (0.45-μm pore size) to remove cell debris and subsequently concentrated via low-speed centrifugation using a 20% sucrose cushion. Virus pellets were resuspended in phosphate-buffered saline (PBS). Generated virus stocks were aliquoted, titrated on confluent BHK-21 monolayers using a plaque assay, and stored at –80°C until use.

### Virus neutralization in human serum.

Normal human serum (NHS) was prepared from blood of 8 to 10 healthy volunteers. Blood was allowed to clot at room temperature and subsequently centrifuged at 800 × *g* for 10 min. Serum was collected and stored in working aliquots at –80°C until use. Some of the aliquots were heat inactivated at 56°C for 30 min and stored at –80°C until it was used as heat-inactivated NHS (hiNHS).

In serum resistance assays, 10^6^ PFU/ml of VSV or VSV-GP in a total volume of 100 μl was incubated in the presence of 50% hiNHS or NHS for 45 min at 37°C in a Thermo-Shaker at 650 rpm. As a control, virus was incubated in GMEM alone (w/o). The remaining infectious virus was determined by a plaque assay on BHK-21 cells. To analyze the kinetic of serum neutralization, samples were assessed after different incubation periods from 5 to 60 min. The effect of serum concentration on VSV-GP neutralization was tested using 10, 20, and 50% concentrations of either hiNHS or NHS. We also investigated neutralization in human serum in the presence of GP-specific antibodies. For this, we performed experiments in the presence of the LCMV GP-specific neutralizing antibody clone Wen4 (in a 1:10 dilution of a hybridoma cell supernatant, kindly provided by Annette Oxenius, ETH Zurich, Switzerland) or an appropriate isotype control (IgG2a). Alternatively, 1:10 diluted heat-inactivated rabbit serum (hiRS) from VSV-GP-immunized rabbits was used as source of GP-specific antibodies. Serum was collected 10 days after the VSV-GP immunization (3 × 10^9^ 50% tissue culture infective doses applied intravenously). Here, we applied heat-inactivated nonimmune rabbit serum as a negative control.

To confirm the role of complement for VSV-GP neutralization in human serum, we blocked complement activation using EDTA (Invitrogen, Thermo Fisher) at concentrations ranging from 0.625 to 10 mM. To further investigate complement pathways involved in neutralization of VSV-GP, experiments were repeated in the presence of MgEGTA (0.1 M MgCl_2_ [Scharlau] and 0.1 M EGTA [Fluka], sterile filtration, pH 7.3), known to selectively inhibit classical and lectin pathways but not alternative pathways of complement activation.

To determine the involvement of natural antibodies of the IgM isotype (nIgM) in the neutralization of VSV-GP, we performed serum resistance assays in the presence of anti-human IgM antibodies (Bethyl Laboratories, Inc.) as described previously (6). NHS, hiNHS, or GMEM were incubated with 10 μg of anti-human IgM antibody for 30 min on ice prior to the addition of virus, and serum resistance assays were conducted as described above.

Natural IgMs against xenoantigens such as the carbohydrate structure galactose-α-1,3-galactose (α-Gal) or nonhuman sialic acid *N*-glycolylneuraminic acid (Neu5Gc) are present in NHS (18, 44). Thus, we incubated NHS or hiNHS with either 10 mM α1-3 galactobiose (Dextra Laboratories) or 10 mM Neu5Gc (A. G. Scientific, Inc.) for 30 min on ice in order to saturate nIgMs recognizing these molecules. We also used 10 mM *N*-acetylneuraminic acid (Neu5Ac; A. G. Scientific, Inc.) as a negative control. Subsequently virus was added, and serum resistance assays were performed as described above.

### Serum stability of virus-producing cell lines.

Different virus-producing cell lines (L929, BHK21, Vero, and A549) were detached with Accutase (Sigma-Aldrich) and washed, and 2 × 10^5^ cells were resuspended in 80 μl of the respective medium. Afterward, the cells were incubated with 20% nonimmune hiNHS or NHS for 1 h at 37°C. The cells were washed and resuspended in PBS supplemented with propidium iodide. The cell viability was analyzed by flow cytometry.

### Virus capture assay.

To detect α-Gal and Neu5Gc on the viral surface, we performed a virus capture assay (VCA) described elsewhere (45). Briefly, 96-well enzyme-linked immunosorbent assay (ELISA) plate was coated overnight at 4°C with polyclonal rabbit anti-mouse immunoglobulins (Dako) diluted in coating buffer (0.1 M NaHCO_3_ [pH 9.6]) at a concentration of 25 μg/ml. The plates were washed with PBS, and specific antibodies (Abs) against the viral envelope (antibody clone KL-25, kindly provided by Anette Oxenius, ETH Zurich, Switzerland) or α-Gal (Enzo) and the corresponding isotype controls (IgG1 and IgM, respectively) were added. Alternatively, ELISA plates were directly coated with a polyclonal chicken IgY Ab against Neu5Gc or the corresponding isotype control (both from BioLegend) at a dilution of 1:25. After 3 h of incubation at 4°C, the plates were washed with PBS, and 10^6^ PFU of VSV-GP(L929) or VSV-GP(Vero) was added to the wells in 50 μl of RPMI medium. After overnight incubation at 4°C, the plates were washed five times with RPMI medium. The RNA of the bound virus was harvested using a virus RNA isolation kit (Qiagen) and amplified by real-time PCR. RNA from the capture assay of VSV-GP was tested in triplicate by using the forward primer 5′-AGTACCGGAGGATTGACGACTAAT-3′, the reverse primer 5′-TCAAACCATCCGAGCCATTC-3′, and the probe 5′-FAM-ACCGCCACAAGGCAGAGATGTGGT-BHQ-3′ (all from Sigma). Reactions were performed with the iTaq Universal Probes one-step kit (Bio-Rad) using a 10-μl reaction mixture volume. The reaction was run on an iCycler iQ (Bio-Rad) at following settings: 10 min at 50°C, 2 min at 95°C, followed by 40 cycles of at 95°C for 15 s and 60°C for 30 s. The data were analyzed using an iCycler iQ data analysis module.

### Transduction of cell lines.

To prove the role of the xenoepitope α-Gal in the complement sensitivity of VSV-GP, we generated a human A549 cell line stably expressing 3Galβ1-GlcNAc-α1-galactosyltransferase (α-GT) and used these cells for virus production. The open reading frame for the porcine α-GT gene (46) was purchased from GeneArt (Thermo Fisher Scientific) and cloned via AgeI/EcoRI sites into the gammaretroviral vector MP91-mcs-IRES-Puro (47). Gammaretroviral particles were produced as described previously (48). A549 cells were transduced with the supernatant and subsequently selected with 10 μg/μl puromycin (Gibco/Thermo Fisher). After several rounds of passaging, α-Gal expression on the surfaces of A549 cells was confirmed by flow cytometry, and the cells were used for VSV-GP production.

### Flow cytometry.

Cells were detached with Accutase (Sigma-Aldrich) and washed two times with PBS supplemented with 2% FCS and 5 mM EDTA. The monoclonal α-Gal antibody clone M86 (EnzoLife Sciences, Inc.) and the secondary anti-mouse IgM-APC (BD Pharmingen) were used to detect α-Gal expression. Samples were measured using a FACSCanto II (Becton Dickinson) flow cytometer and analyzed using FlowJo software (FlowJo).

### Statistical analysis.

All statistical analyses were performed with Prism 5 software (GraphPad) using an ordinary one-way analysis of variance (ANOVA), followed by Dunnett’s multiple-comparison test. Further information on the *P* values is given in the figure legends.

## References

[1] Marelli G, Howells A, Lemoine NR, Wang Y 2018 Oncolytic viral therapy and the immune system: a double-edged sword against cancer. Front Immunol 9:1–8. 29755464 10.3389/fimmu.2018.00866 PMC5932159

[2] Melzer M, Lopez-Martinez A, Altomonte J 2017 Oncolytic vesicular stomatitis virus as a viro-immunotherapy: defeating cancer with a “hammer” and “anvil.” Biomedicines 5:8. doi:10.3390/biomedicines5010008. PMC5423493 28536351

[3] Zemp F, Rajwani J, Mahoney DJ 2018 Rhabdoviruses as vaccine platforms for infectious disease and cancer. Biotechnol Genet Eng Rev 34:122–138. doi:10.1080/02648725.2018.1474320. 29781359

[4] Bachmann MF, Odermatt B, Hengartner H, Zinkernagel RM 1996 Induction of long-lived germinal centers associated with persisting antigen after viral infection. J Exp Med 183:2259–2269. doi:10.1084/jem.183.5.2259. 8642335 PMC2192556

[5] Beebe DP, Cooper NR 1981 Neutralization of vesicular stomatitis virus (VSV) by human complement requires a natural IgM antibody present in human. J Immunol 126:1562–1568. 6259260

[6] Tesfay MZ, Ammayappan A, Federspiel MJ, Barber GN, Stojdl D, Peng K-W, Russell SJ 2014 Vesiculovirus neutralization by natural IgM and complement. J Virol 88:6148–6157. doi:10.1128/JVI.00074-14. 24648451 PMC4093862

[7] Stoermer KA, Morrison TE 2011 Complement and viral pathogenesis. Virology 411:362–373. doi:10.1016/j.virol.2010.12.045. 21292294 PMC3073741

[8] van den Pol AN, Dalton KP, Rose JK 2002 Relative neurotropism of a recombinant rhabdovirus expressing a green fluorescent envelope glycoprotein. J Virol 76:1309–1327. doi:10.1128/JVI.76.3.1309-1327.2002. 11773406 PMC135838

[9] Muik A, Kneiske I, Werbizki M, Wilflingseder D, Giroglou T, Ebert O, Kraft A, Dietrich U, Zimmer G, Momma S, von Laer D 2011 Pseudotyping vesicular stomatitis virus with lymphocytic choriomeningitis virus glycoproteins enhances infectivity for glioma cells and minimizes neurotropism. J Virol 85:5679–5684. doi:10.1128/JVI.02511-10. 21450833 PMC3094995

[10] Muik A, Stubbert LJ, Jahedi RZ, Geiss Y, Kimpel J, Dold C, Tober R, Volk A, Klein S, Dietrich U, Yadollahi B, Falls T, Miletic H, Stojdl D, Bell JC, von Laer D 2014 Re-engineering vesicular stomatitis virus to abrogate neurotoxicity, circumvent humoral immunity, and enhance oncolytic potency. Cancer Res 74:3567–3578. doi:10.1158/0008-5472.CAN-13-3306. 24812275

[11] Tober R, Banki Z, Egerer L, Muik A, Behmuller S, Kreppel F, Greczmiel U, Oxenius A, von Laer D, Kimpel J 2014 VSV-GP: a potent viral vaccine vector that boosts the immune response upon repeated applications. J Virol 88:4897–4907. doi:10.1128/JVI.03276-13. 24554655 PMC3993835

[12] Forsgren A, Quie PG 1974 Opsonic activity in human serum chelated with ethylene glycoltetra-acetic acid. Immunology 26:1251–1256. 4604857 PMC1423361

[13] Fine DP 1977 Comparison of ethyleneglycoltetraacetic acid and its magnesium salt as reagents for studying alternative complement pathway function. Infect Immun 16:124–128. 406201 10.1128/iai.16.1.124-128.1977 PMC421498

[14] Schiela B, Bernklau S, Malekshahi Z, Deutschmann D, Koske I, Banki Z, Thielens NM, Würzner R, Speth C, Weiss G, Stiasny K, Steinmann E, Stoiber H 2018 Active human complement reduces the Zika virus load via formation of the membrane-attack complex. Front Immunol 9:2177. doi:10.3389/fimmu.2018.02177. 30386325 PMC6199351

[15] Galili U, Macher BA, Buehler J, Shohet SB 1985 Human natural anti-α-galactosyl IgG. Biochim Biophys Acta 162:573–582. doi:10.1084/jem.162.2.573. PMC2187733 2410529

[16] Hamadeh RM, Galili U, Zhou P, Griffiss JM 1995 Anti-α-galactosyl immunoglobulin A (IgA), IgG, and IgM in human secretions. Clin Diagn Lab Immunol 2:125–131. 7697518 10.1128/cdli.2.2.125-131.1995 PMC170114

[17] Galili U, Shohet SB, Kobrin E, Stults CL, Macher BA 1988 Man, apes, and Old World monkeys differ from other mammals in the expression of alpha-galactosyl epitopes on nucleated cells. J Biol Chem 263:17755–17762. 2460463

[18] Zhu A, Hurst R 2002 Anti-*N*-glycolylneuraminic acid antibodies identified in healthy human serum. Xenotransplantation 9:376–381. doi:10.1034/j.1399-3089.2002.02138.x. 12371933

[19] Varki A 2001 Loss of N-glycolylneuraminic acid in humans: mechanisms, consequences, and implications for hominid evolution. Am J Phys Anthropol 116:54–69. doi:10.1002/ajpa.10018. PMC7159735 11786991

[20] Eschli B, Zellweger RM, Wepf A, Lang KS, Quirin K, Weber J, Zinkernagel RM, Hengartner H 2007 Early antibodies specific for the neutralizing epitope on the receptor binding subunit of the lymphocytic choriomeningitis virus glycoprotein fail to neutralize the virus. J Virol 81:11650–11657. doi:10.1128/JVI.00955-07. 17699567 PMC2168768

[21] Dold C, Rodriguez Urbiola C, Wollmann G, Egerer L, Muik A, Bellmann L, Fiegl H, Marth C, Kimpel J, von Laer D 2016 Application of interferon modulators to overcome partial resistance of human ovarian cancers to VSV-GP oncolytic viral therapy. Mol Ther Oncolytics 3:16021. doi:10.1038/mto.2016.21. 27738655 PMC5040171

[22] Kimpel J, Urbiola C, Koske I, Tober R, Banki Z, Wollmann G, von Laer D 2018 The oncolytic virus VSV-GP is effective against malignant melanoma. Viruses 10:108. doi:10.3390/v10030108. PMC5869501 29498639

[23] Humphreys IR, Sebastian S 2018 Novel viral vectors in infectious diseases. Immunology 153:1–9. doi:10.1111/imm.12829. 28869761 PMC5721250

[24] Ikeda K, Wakimoto H, Ichikawa T, Jhung S, Hochberg FH, Louis DN, Chiocca EA 2000 Complement depletion facilitates the infection of multiple brain tumors by an intravascular, replication-conditional herpes simplex virus mutant. J Virol 74:4765–4775. doi:10.1128/jvi.74.10.4765-4775.2000. 10775615 PMC111999

[25] Evgin L, Ilkow CS, Bourgeois-Daigneault M-C, de Souza CT, Stubbert L, Huh MS, Jennings VA, Marguerie M, Acuna SA, Keller BA, Lefebvre C, Falls T, Le Boeuf F, Auer RA, Lambris JD, McCart JA, Stojdl DF, Bell JC 2016 Complement inhibition enables tumor delivery of LCMV glycoprotein pseudotyped viruses in the presence of antiviral antibodies. Mol Ther Oncolytics 3:16027. doi:10.1038/mto.2016.27. 27909702 PMC5111574

[26] Diebolder CA, Beurskens FJ, De Jong RN, Koning RI, Strumane K, Lindorfer MA, Voorhorst M, Ugurlar D, Rosati S, Heck AJR, Van De Winkel JGJ, Wilson IA, Koster AJ, Taylor RP, Saphire EO, Burton DR, Schuurman J, Gros P, Parren P 2014 Supplementary materials for complement is activated by IgG hexamers assembled at the cell surface. Science 14:1260–1264. doi:10.1126/science.1248943. PMC4250092 24626930

[27] Panda S, Ding JL 2015 Natural antibodies bridge innate and adaptive immunity. J Immunol 194:13–20. doi:10.4049/jimmunol.1400844. 25527792

[28] Johnson JB, Lyles DS, Alexander-Miller MA, Parks GD 2012 Virion-associated complement regulator CD55 is more potent than CD46 in mediating resistance of mumps virus and vesicular stomatitis virus to neutralization. J Virol 86:9929–9940. doi:10.1128/JVI.01154-12. 22761385 PMC3446622

[29] Welsch S, Müller B, Kräusslich HG 2007 More than one door: budding of enveloped viruses through cellular membranes. FEBS Lett 581:2089–2097. doi:10.1016/j.febslet.2007.03.060. 17434167 PMC7126970

[30] Galili U 2013 Anti-Gal: An abundant human natural antibody of multiple pathogeneses and clinical benefits. Immunology 140:1–11. doi:10.1111/imm.12110. PMC3809700 23578170

[31] Goochee CF, Gramer MJ, Andersen DC, Bahr JB, Rasmussen JR 1991 The oligosaccharides of glycoproteins: bioprocess factors affecting oligosaccharide structure and their effect on glycoprotein properties. Nat Biotechnol 9. doi:10.1038/nbt1291-1347. 1367768

[32] Rother RP, Fodor WL, Springhorn JP, Birks CW, Setter E, Sandrin MS, Squinto SP, Rollins SA 1995 A novel mechanism of retrovirus inactivation in human serum mediated by anti-α-galactosyl natural antibody. J Exp Med 182:1345–1355. doi:10.1084/jem.182.5.1345. 7595205 PMC2192220

[33] Galili U 2012 Induced anti-non Gal antibodies in human xenograft recipients. Transplantation 93:11–16. doi:10.1097/TP.0b013e31823be870. 22146315

[34] Bresk CA, Hofer T, Wilmschen S, Krismer M, Beierfuß A, Effantin G, Weissenhorn W, Hogan MJ, Jordan APO, Gelman RS, Montefiori DC, Liao HX, Schmitz JE, Haynes BF, von Laer D, Kimpel J 2019 Induction of tier 1 HIV neutralizing antibodies by envelope trimers incorporated into a replication competent vesicular stomatitis virus vector. Viruses 11:159. doi:10.3390/v11020159. PMC6409518 30769947

[35] Ong GL, Mattes MJ 1989 Mouse strains with typical mammalian levels of complement activity. J Immunol Methods 125:147–158. doi:10.1016/0022-1759(89)90088-4. 2607149

[36] Spear GT, Lurain NS, Parker CJ, Ghassemi M, Payne GH, Saifuddin M 1995 Host cell-derived complement control proteins CD55 and CD59 are incorporated into the virions of two unrelated enveloped viruses: human T cell leukemia/lymphoma virus type I (HTLV-I) and human cytomegalovirus (HCMV). J Immunol 155:4376 LP–4381. 7594597

[37] Stoiber H, Pintér C, Siccardi AG, Clivio A, Dierich MP 1996 Efficient destruction of human immunodeficiency virus in human serum by inhibiting the protective action of complement factor H and decay accelerating factor (DAF, CD55). J Exp Med 183:307–310. doi:10.1084/jem.183.1.307. 8551237 PMC2192395

[38] Croyle MA, Callahan SM, Auricchio A, Schumer G, Linse KD, Wilson JM, Brunner LJ, Kobinger GP 2004 PEGylation of a vesicular stomatitis virus G pseudotyped lentivirus vector prevents inactivation in serum. J Virol 78:912–921. doi:10.1128/JVI.78.2.912-921.2004. 14694122 PMC368741

[39] Hwang B-Y, Schaffer DV 2013 Engineering a serum-resistant and thermostable vesicular stomatitis virus G glycoprotein for pseudotyping retroviral and lentiviral vectors. Gene Ther 20:807–815. doi:10.1038/gt.2013.1. 23364315 PMC3735647

[40] Schauber-Plewa C, Simmons A, Tuerk MJ, Pacheco CD, Veres G 2005 Complement regulatory proteins are incorporated into lentiviral vectors and protect particles against complement inactivation. Gene Ther 12:238–245. doi:10.1038/sj.gt.3302399. 15550926

[41] Wakimoto H, Ikeda K, Abe T, Ichikawa T, Hochberg FH, Ezekowitz RAB, Pasternack MS, Chiocca EA 2002 The complement response against an oncolytic virus is species-specific in its activation pathways. Mol Ther 5:275–282. doi:10.1006/mthe.2002.0547. 11863417

[42] Evgin L, Acuna SA, Tanese De Souza C, Marguerie M, Lemay CG, Ilkow CS, Findlay CS, Falls T, Parato KA, Hanwell D, Goldstein A, Lopez R, Lafrance S, Breitbach CJ, Kirn D, Atkins H, Auer RC, Thurman JM, Stahl GL, Lambris JD, Bell JC, Mccart JA 2015 Complement inhibition prevents oncolytic vaccinia virus neutralization in immune humans and cynomolgus macaques. Mol Ther 23:1066–1076. doi:10.1038/mt.2015.49. 25807289 PMC4817751

[43] Abdel-Motal UM, Wang S, Awad A, Lu S, Wigglesworth K, Galili U 2010 Increased immunogenicity of HIV-1 p24 and gp120 following immunization with gp120/p24 fusion protein vaccine expressing alpha-Gal epitopes. Vaccine 28:1758–1765. doi:10.1016/j.vaccine.2009.12.015. 20034607 PMC2866524

[44] Welsh RM, O’Donnell CL, Reed DJ, Rother RP 1998 Evaluation of the Galα 1-3Gal epitope as a host modification factor eliciting natural humoral immunity to enveloped viruses. J Virol 72:4650–4656. 9573228 10.1128/jvi.72.6.4650-4656.1998 PMC109985

[45] Ejaz A, Steinmann E, Bánki Z, Anggakusuma, Khalid S, Lengauer S, Wilhelm C, Zoller H, Schloegl A, Steinmann J, Grabski E, Kleines M, Pietschmann T, Stoiber H 2012 Specific acquisition of functional CD59 but not CD46 or CD55 by hepatitis C virus. PLoS One 7:e45770. doi:10.1371/journal.pone.0045770. 23049856 PMC3458075

[46] Strahan K, Gu F, Preece A, Gustavsson I, Andersson L, Gustafsson K 1995 cDNA sequence and chromosome localization of pig α1,3 galactosyltransferase. Immunogenetics 41. doi:10.1007/BF00182319. 7528726

[47] Miletic H, Fischer Y, Litwak S, Giroglou T, Waerzeggers Y, Winkeler A, Li H, Himmelreich U, Lange C, Stenzel W, Deckert M, Neumann H, Jacobs AH, von Laer D 2007 Bystander killing of malignant glioma by bone marrow-derived tumor-infiltrating progenitor cells expressing a suicide gene. Mol Ther 15:1373–1381. doi:10.1038/sj.mt.6300155. 17457322

[48] Hermann FG, Egerer L, Brauer F, Gerum C, Schwalbe H, Dietrich U, von Laer D 2009 Mutations in gp120 contribute to the resistance of human immunodeficiency virus type 1 to membrane-anchored C-peptide maC46. J Virol 83:4844–4853. doi:10.1128/JVI.00666-08. 19279116 PMC2682112

[49] Hayashi S, Ogawa S, Takashima Y, Otsuka H 2004 The neutralization of pseudorabies virus by anti-α-galactocyl natural antibody in normal serum. Virus Res 99:1–7. doi:10.1016/j.virusres.2003.09.008. 14687940

